# Increasing the performance of the bullet core by changing the geometry. an experimental and numerical study

**DOI:** 10.1007/s12024-025-01024-5

**Published:** 2025-06-13

**Authors:** Ender Çelik, Ali Koç

**Affiliations:** Department of Mechanical Engineering İskenderun/Hatay, Iskenderun Technical University, İskenderun, Turkey

**Keywords:** Fluted Bullet, 9 × 19 Luger Bullet, Ballistic Gelatin, Ansys, FEM

## Abstract

This study aims to transfer the entire kinetic energy of a geometrically unique 9 × 19 mm bullet onto the target without compromising its penetration capability. In an era where asymmetric security threats are increasingly prevalent, the newly designed bullet core is proposed as a potential "force multiplier" for security forces. The ANSYS Explicit Dynamics finite element model was employed to investigate the penetration behaviors of both the newly designed and currently used 9 × 19 mm bullets into human tissue analogs. %10 ballistic gelatin was utilized to simulate human tissue in the experiments. In this paper, the penetration effects of the newly designed and in-service 9 × 19 mm bullets on ballistic gelatin were first numerically modeled using finite element methods (FEM). The numerical findings were subsequently validated through experimental testing. Results indicated that the bullet featuring the new geometric design produced greater damage to the ballistic gelatin compared to the conventional design.

## Highlights

• Small arms chambered in 9 × 19 mm are the most widely utilized self-defense weapons worldwide.

• The 9 × 19 mm Luger cartridge has undergone minimal changes since its original design.

• A redesigned 9 × 19 mm bullet core was developed to enhance tissue damage while maintaining effective penetration.

• Experimental tests demonstrated that the new bullet configuration significantly increases tissue damage without compromising penetration capability.

## Introduction

Recently, there has been an increasing demand for ammunition used in military operations, training exercises, and related applications. The majority of armed encounters involving law enforcement personnel occur within a 7-m range. During such engagements, law enforcement agencies predominantly utilize 9 × 19 mm small arms. Although these bullets have been in service for many years, their effectiveness in reliably incapacitating suspects has been subject to ongoing scrutiny. Consequently, various shooting techniques have been developed to mitigate this limitation. This study aims to design a new bullet core geometry that is compatible with the small arms used by law enforcement during close-range engagements, capable of causing greater tissue damage without compromising penetration capability. The damage inflicted by the newly designed bullet core will be analyzed numerically using the finite element method (FEM) within the ANSYS software environment. Human tissue will be simulated using 10% ballistic gelatin with equivalent mechanical properties. Finally, the numerical analyses will be validated through experimental testing.

The 9 × 19 mm Parabellum bullet was first developed by George Luger in Germany in mid-1901 [1]. In early 1902, through Vickers Limited, Luger proposed a 9 × 19 mm variant of the 7.65 × 21 mm pistol to the Small Arms Committee. By mid-1903, three prototype 9 × 19 mm Luger pistols were delivered to the U.S. Army for testing at the Springfield Arsenal, marking the first documented use of 9 × 19 mm ammunition [2]. Since then, it has been recognized as the most widely used pistol and submachine gun ammunition, including within NATO countries, due to its low cost and ease of supply [3].

Several studies have examined various aspects of bullet behavior. Khan and Saha numerically analyzed the aerodynamic effects of airflow around bullets with different nose geometries, evaluating the distribution of velocity and pressure around sharp-edged and rounded profiles [4]. Selimli conducted numerical simulations to investigate the aerodynamic behavior of 9 × 19 mm bullets by modifying their surface profiles [5]. Widyastuti et al. employed a finite element model with a Lagrangian frame and a Lagrangian-discrete element method (DEM) to study the deformation and penetration characteristics of frangible bullets in ballistic gelatin [6]. Gilson et al. performed experimental and numerical investigations into the ballistic response of gelatin blocks protected by composite plates. Using high-speed cameras and pressure gauges, they recorded the evolution of the temporary cavity and transient pressures, demonstrating good agreement between experimental observations and numerical predictions. Their study revealed multiple pressure waves propagating through the gelatin, and they compared the damage mechanisms observed in both bullets and protective materials [7]. Riva et al. analyzed the trajectories of 9 × 19 mm bullets fired into ballistic gelatin from various distances and angles, observing consistent trajectory patterns across tests [8]. Jin et al. conducted extensive experimental and numerical studies on the penetration of BB-gun pellets into a"cowhide + gelatin"composite target to better understand BB-gun-related injuries. Their findings confirmed that BB-gun pellets can cause serious or fatal injuries to vital organs and proposed a dimensional analysis-based model to predict ballistic limits based on pellet properties. [9] Schyma et al. measured the velocity loss of 9 × 19 mm bullets traveling through ballistic gelatin, concluding that the deceleration rate of deformed bullets remained constant [10]. Meng et al. modeled 10% ballistic gelatin using the Smoothed Particle Hydrodynamics (SPH) method to observe micro-level reactions within the gelatin under ballistic impact conditions [11].

The present study falls within the field of wound (terminal) ballistics, one of the four primary domains of ballistic research. Numerous studies investigating wound ballistics have been reported in the literature [12–19]. Bullet penetration behavior is influenced by several factors, including bullet type, rotational and translational velocities, and impact angle.

Investigating wound ballistics presents several challenges. One of the primary difficulties lies in the fact that these events occur over an extremely short timescale (on the order of a few microseconds) and exhibit highly complex behaviors that are nearly impossible to replicate in opaque biological materials such as blood and skin. To address this issue, transparent ballistic gelatin blocks—a pseudoelastic or superelastic material—or plastic mediums such as soap are employed. These materials not only possess mechanical properties similar to human tissues but also allow visualization of internal deformation. The use of high-speed cameras is essential to capture the rapid and extensive deformations that occur during ballistic events. Upon impact, the bullet immediately undergoes swirling, yawing, and tumbling motions due to the resistive forces encountered, mirroring the behavior observed during penetration into human tissue. Achieving physically meaningful numerical simulations of such highly nonlinear, velocity-dependent penetration phenomena within extremely short timescales remains a significant challenge. In this study, real firing tests were conducted to validate the finite element (FE) simulations. For the numerical modeling, the Equation of State (EOS), the Johnson–Cook failure model, and a viscoelastic material model were implemented to represent the behaviors of the bullet and the ballistic gelatin.

In this study, particular emphasis is placed on the penetration behavior of two different bullets, focusing on their movement and trajectories within ballistic gelatin (BG), as well as the formation of temporary and permanent cavities in BG. The investigation is structured as follows:

- Sec."Materials and methods"provides a brief explanation of material failure models and material properties, along with the definitions of finite element analysis procedures, material modeling approaches, and boundary conditions.
- Sec."Numerical analyses and experiments"presents a comparison between the results of shooting tests and finite element (FE) simulations.
- The conclusion section summarizes the key findings and proposes directions for future research.

## Materials and methods

### What is FEM

**Fig. 1:**
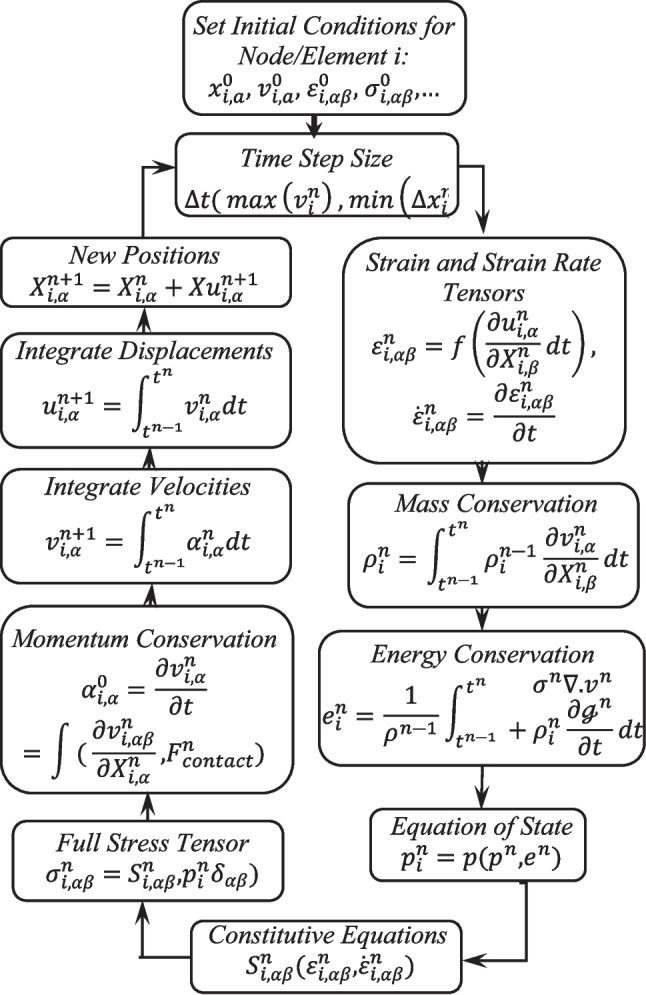
Finite element solution procedure [23]

The finite element method (FEM) is a widely recognized and reliable technique employed in numerical analysis and engineering design [20–22]. Based on the principle of progressing"from part to whole,"FEM is used to model specific regions or components of two- and three-dimensional structures. Initially developed for aerospace engineering applications in the 1950 s, FEM expanded to other engineering disciplines in the 1970 s with technological advancements, including mechanical, electrical, aeronautical, civil, hydrodynamic, and biomedical engineering. Due to the complexity of real-world geometries, simplified elements are used to discretize structures within a computational environment, and the laws of physics are applied to these individual elements. A mesh network is employed to divide the entire structure into elements, with nodes defined at the intersection points of these elements. Increasing the number of elements enhances the precision of force distribution calculations. The x, y, and z coordinates of each node are determined and input into the computational model, and the material properties of the elements are assigned accordingly. In the mathematical model, matrices representing state changes are constructed by applying external loads and boundary conditions to the nodes, and these matrices are subsequently solved using computational methods. Through this process, the stresses and strains at each element, as well as the overall distribution within the entire structure, can be obtained. Among various computational tools available for wound ballistics analysis, this study utilized the ANSYS Explicit Dynamics program to solve the mass conservation equation, the energy conservation equation, and the equation of state (EOS), as illustrated in Fig. 1.

The equation of state (EOS) is employed to characterize the hydrodynamic response of solid materials under dynamic loading conditions. In fluids such as gases or liquids, which do not sustain shear stresses, the internal pressure is solely a function of density and internal energy. Under conditions of high-speed deformation, solid materials exhibit fluid-like behavior, allowing their internal pressures to be effectively calculated using the equation of state [23]. Due to the rapid deformation characteristics of ballistic gelatin, the Mie–Grüneisen EOS is utilized. The Mie–Grüneisen EOS is essentially a Taylor series approximation based on empirical Hugoniot data; however, it is known that this EOS does not account for phase transitions [24].

### The bullet cores

**Fig. 2:**
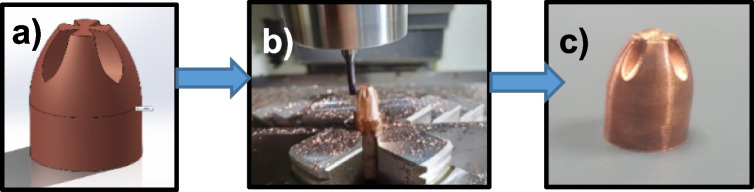
Bullet manufacturing process in new geometry

The 9 × 19 mm Luger bullet core traditionally consists of a brass jacket and a lead inner core, designed with aerodynamic geometry. This configuration enhances the projectile's ability to penetrate targets while preserving initial velocity and flight stability. Additionally, the density and geometric shape of the core material ensure structural integrity and contribute significantly to improved penetration performance. The newly developed bullet core, presented in this study, incorporates an innovative geometric design intended to maximize energy transfer upon impact with the target surface (Fig. 2). This redesigned shape substantially increases tissue damage upon impact. Moreover, its carefully engineered geometry maintains weight balance, improves destructive efficiency, and optimizes recoil management and aerodynamic stability. Conventional 9 × 19 mm Luger ammunition frequently utilizes hazardous materials such as lead (Pb), posing considerable health risks to users and negative environmental impacts [25, 26]. Consequently, copper was selected as the primary material for manufacturing the redesigned bullet core. As a result, the new design enhances operational reliability and effectiveness while significantly improving stopping power against suspects. Ultimately, this bullet design aims to assist law enforcement personnel in executing their duties more effectively and enhancing public safety.

### Ballistic gelatin

Ballistic gelatin is a test medium specifically developed to replicate the effects of projectile wounds on muscle tissue. This medium was initially developed and refined by Martin Fackler and colleagues in the field of wound ballistics to closely resemble human muscle tissue. Typically, ballistic gelatin consists of gelatin powder dissolved in water to create a gel-like material. It effectively simulates the density and viscosity of human and animal muscle tissues and is widely accepted as a standardized medium for evaluating the terminal performance of firearm ammunition. While ballistic gelatin does not entirely reproduce the tensile strength and structural complexities of biological tissues such as skin, muscle, and bone, it provides a highly reliable simulation of soft tissue behavior, yielding comparable outcomes in most ballistic assessments. However, its accuracy may diminish when used to model interactions involving very low-velocity projectiles. The primary advantage of ballistic gelatin over actual muscle tissue lies in its controlled, reproducible properties, allowing consistent and dependable comparisons of terminal ballistic performance (Table 1).

**Table 1:**
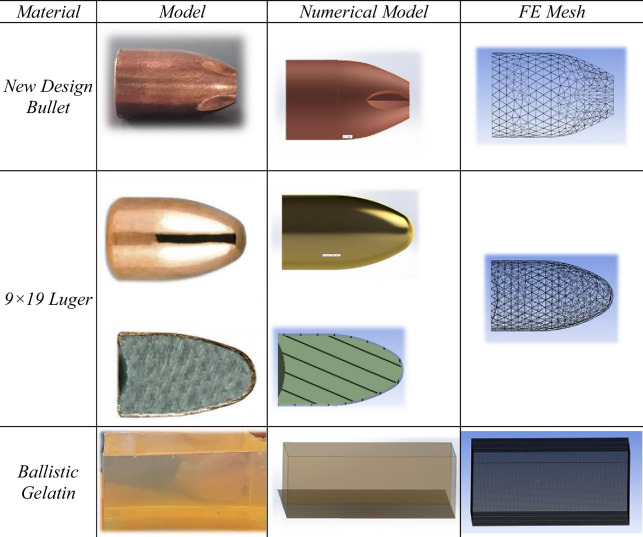
Materials, models, numerical models

### Numerical modeling of materials

In this study, finite element (FE) analyses were performed using the ANSYS Explicit Dynamics software. For accurate FEM simulations, it is essential to numerically define all materials involved and specify appropriate boundary conditions, including material mechanical properties, initial velocities, and stabilization criteria. Accordingly, ballistic gelatin (BG) and bullet cores were numerically modeled (Table 2). The dimensions of the BG model were defined as 15 × 15 × 40 cm, with the mechanical properties of the BG material provided in Table 3.

**Table 2:** Mechanical properties of lead [23]

Density	Steinberg Guinan strength		Shear modulus	Gruneisen coefficient	${c}_{1}$	${s}_{1}$	${s}_{1}$
	Initial yield stress	Maximum yield stress					
1134 kg/m^3^	8 MPa	0.1 Gpa	11.13 Gpa	2.74	2006 ${m.s}^{-1}$	1.429	$0{ m.s}^{-1}$

**Table 3:** Mechanical properties of 10% ballistic gelatin [23]

Density	Viscoelasticity						Mie-Gruneisen EOS (Shock EOS linear)						
	Instantaneous shear modulus		Shear modulus		Viscoelastic decay constant		Gruneisen coefficient			${c}_{1}$	${s}_{1}$		${s}_{2}$
1030 kg/m^3^	0.214 MPa		0.158 MPa		0.00087 ${s}^{-1}$		0.17			1553 m/s	1.93		0 m/s
Johnson–Cook failure model													
${D}_{1}$		${D}_{2}$		${D}_{3}$		${D}_{4}$		${D}_{5}$	Melting temperature			Reference strain rate	
−13549		0.6015		0.2589		0.03012		0	20 °C			0.001	

The conventional 9 × 19 mm Luger bullet core was numerically modeled with an outer brass jacket and an inner lead core, whereas the newly designed 9 × 19 mm bullet core was modeled entirely from copper. Considering the relatively high hardness and high-velocity impact characteristics of the bullet cores, elastoplastic material models with appropriate limit values were applied. Numerical modeling details of the bullet cores are summarized in Table 1, and the mechanical properties of the materials used are presented in Tables 2 and 4.

**Table 4:** Mechanical properties of copper [23]

Density	Bilinear isotropic hardening		Shear modulus	Gruneisen coefficient	${c}_{1}$	${s}_{1}$	${s}_{2}$
	Yield strength	Tangent modulus					
8900 kg/m^3^	70 MPa	2 Gpa	46.4 Gpa	2	3958 ${m.s}^{-1}$	1.497	$0{ m.s}^{-1}$

## Numerical analyses and experiments

### FEA numerical analyzes

**Fig. 3:**
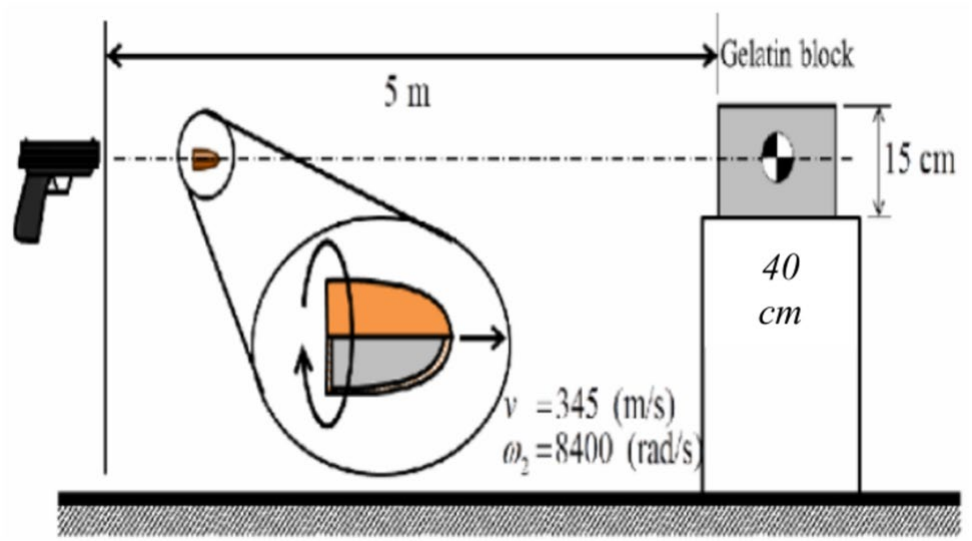
The experimental setup

This section presents the findings obtained from both experimental and numerical analyses addressing rapid deformation phenomena in wound ballistics. In the experimental procedure, ballistic gelatin (BG) blocks were shot with two different bullets using a gas-operated firing mechanism positioned at a distance of 5 m. A high-speed camera capable of capturing up to 50,000 frames per second was employed to observe the bullet cores'trajectories within the BG and to analyze the formation and evolution of temporary and permanent cavities. A schematic representation of the experimental setup is provided in Fig. 3.

The movements of the bullets within the ballistic gelatin (BG) were recorded throughout the experiment using a high-speed camera. The finite element (FE) analysis results were subsequently compared with these experimental observations. Due to the inherently chaotic nature of wound ballistics, achieving exact correspondence between experimental and numerical results is extremely challenging. Nevertheless, a comparative assessment revealed strong similarities between the experimental and FE results in terms of both the trajectories of the bullet cores and the extent of tissue damage observed. The velocity and motion of the projectiles after exiting the BG were not examined, as they were beyond the scope of this study. Upon impact, a projectile in a statically balanced state encounters tissues of varying densities and elasticities. As the projectile transitions from air—a relatively low-density medium—into high-density tissue (simulated by BG), it undergoes a rapid decrease in linear velocity and kinetic energy. This abrupt deceleration, combined with the projectile’s initial angular velocity, causes it to revert toward its hyperstatic equilibrium state [27]. As a result of this sudden deceleration, a deflection of approximately 2° to 4° occurs along the projectile axis, which is sufficient to induce wobbling within the tissue [28]. Upon contact with tissue, the projectile generates pressure waves, resulting in the loss of approximately 99% of its initial kinetic energy. The increasing pressure forces surrounding tissues to displace outward from the center of the cavity. This displacement forms a temporary cavity, which persists during the penetration process. Within milliseconds, the surrounding tissues recoil toward the cavity center, ultimately forming a permanent cavity [29]. Notably, the temporary cavity formations observed in the experimental high-speed recordings exhibited strong agreement with the finite element (FE) simulation results.

Initially, the penetration behavior of each bullet type was simulated through numerical analyses, based on the characteristics of the bullet and the material properties of the target medium. The simulations produced time-dependent profiles of penetration depth for each bullet type, as well as visualizations of the temporary cavity formations surrounding the bullets.

**Fig. 4:**
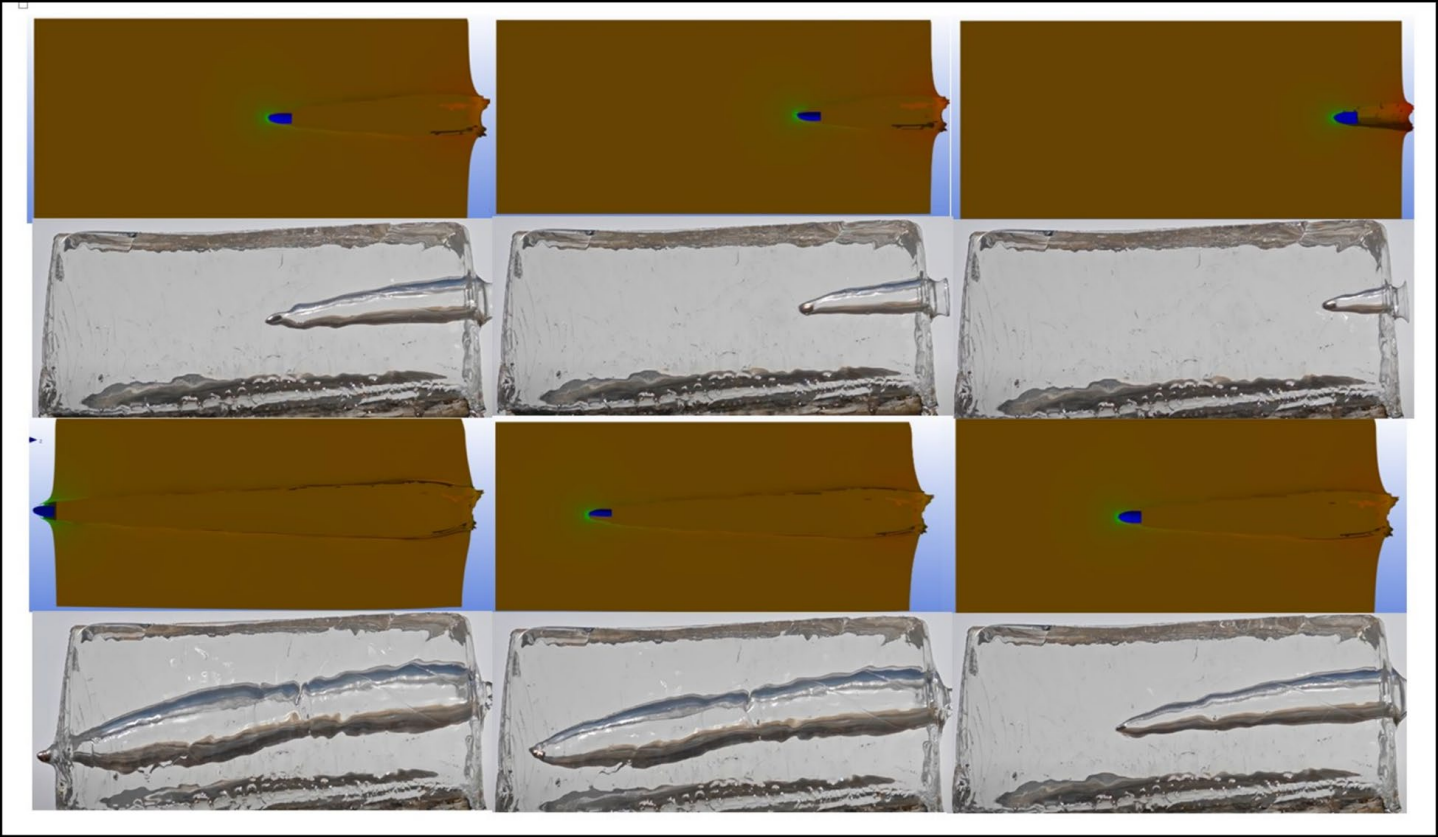
Comparisons of the deformations from the experiment and the finite element simulation for 9 × 19 fmj

**Fig. 5:**
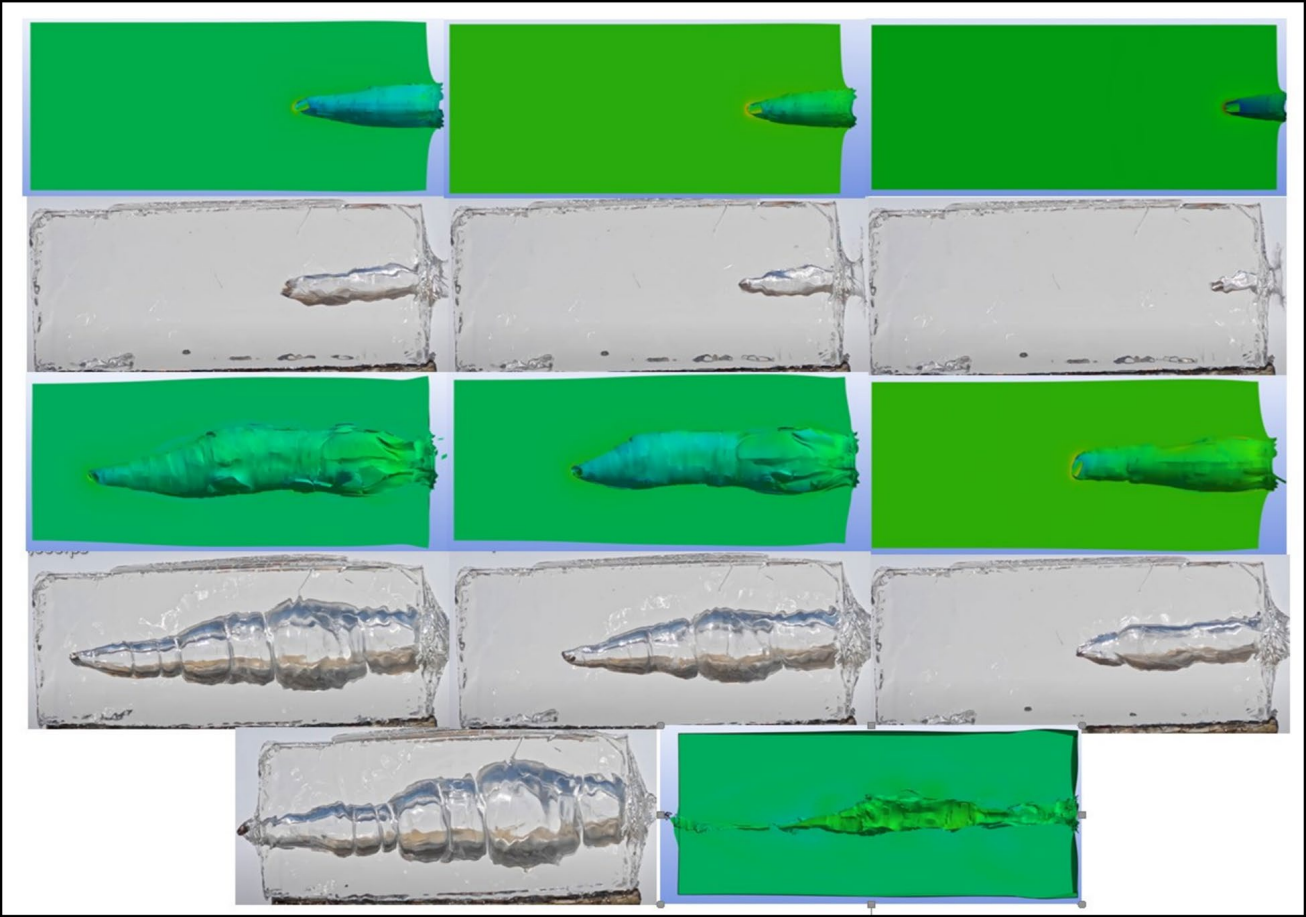
Comparisons of the deformations from the experiment and the finite element simulation for newly designed bullet

Subsequently, these simulation results were validated through controlled experimental studies. Real-world firing tests were conducted to observe the ballistic behavior of the bullets under standardized conditions. The experimental data were then compared with the numerical analysis results, completing the validation process (Figs. 4 and 5).

**Fig. 6:**
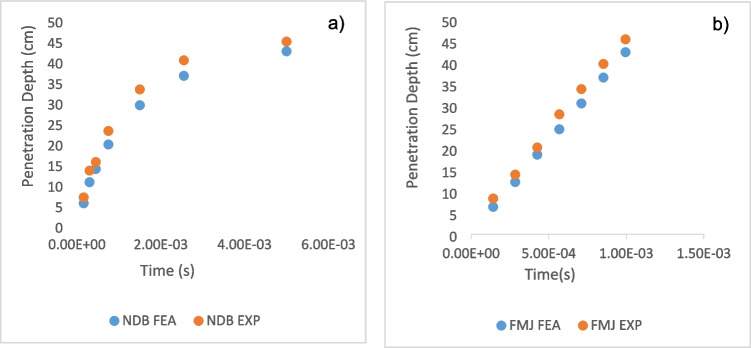
Temporal cavity comparison of bullet cores. **a** 9 × 19 mm FMJ bullet FEA and experimental result **b** Newly designed bullet (NDB) FEA and experimental result

In line with the findings, graphs illustrating the variation of penetration depth over time for each bullet type are presented. By comparing the numerical and experimental results, these graphs clearly demonstrate the influence of bullet type on penetration behavior over time (Fig. 6).

**Fig. 7:**
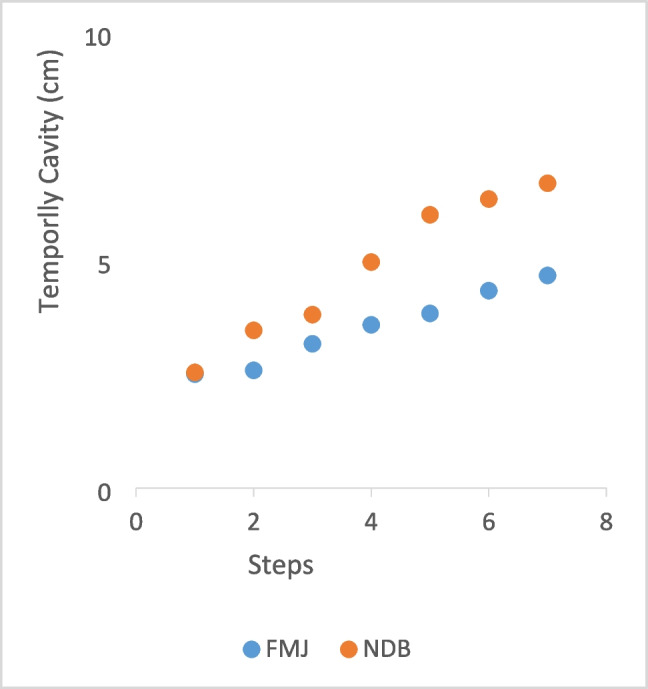
Temporal cavity comparison of bullet cores

Through this integrated analysis, we aim to provide readers with a comprehensive theoretical and practical understanding of how bullet performance evolves over time (Fig. 7).

A temporary cavity refers to a transient cavity that forms and subsequently expands when a bullet or other high-velocity object impacts a material. This phenomenon results from the rapid transfer of kinetic energy at the moment of impact, causing substantial damage to the surrounding medium.

The formation of temporary cavities is a critical area of research in wound ballistics and impact engineering, as it directly affects the durability and failure mechanisms of the impacted material. Understanding the effects of temporary cavity formation on biological tissues is essential for both injury analysis and the design of protective systems. These effects can be broadly summarized as follows:

Formation Mechanism: A temporary cavity forms when a bullet or projectile contacts body tissues. During this brief interaction, a portion of the projectile’s kinetic energy concentrates at the point of impact, resulting in a sudden increase in pressure and the formation of a transient cavity.

Expansion and Effects: The initial temporary cavity, formed at the moment of impact, expands outward from a localized region. This expansion process can deform surrounding tissues, particularly affecting soft tissues and bones by inducing plastic deformation or causing a transition from elastic to plastic behavior.

Destructive Potential: The expansion of the temporary cavity can result in significant damage to surrounding tissues. Under the influence of high-velocity projectiles, tissues subjected to the forces of the temporary cavity may experience cracking, fragmentation, or internal hemorrhaging. Understanding this destructive potential is crucial for a comprehensive assessment of ballistic injuries and post-traumatic effects.

Applications and Importance: Temporary cavity formation plays a critical role in the design of ballistic protection systems and the clinical management of ballistic injuries. Accurate characterization of this phenomenon is vital for military personnel, law enforcement agencies, and researchers in the field of ballistic protection dealing with projectile impacts.

The effects of temporary cavity formation contribute to advancements in medical treatment technologies, materials science, and impact engineering. An in-depth understanding of this phenomenon, coupled with the development of effective prevention and treatment strategies, enables the design of more effective future security and medical solutions. A comparison of the two bullet designs with respect to this critical parameter is presented in Fig. 6.

## Conclusions

In this study, experimental and numerical investigations were conducted on viscoelastic ballistic gelatin to evaluate the effects of high-speed collisions between a widely used 9 × 19 mm full metal jacket (FMJ) bullet and a newly designed bullet. Finite element analysis (FEA) was performed using the ANSYS Workbench platform. The impact effects of the two different bullet types on human tissue analogs were systematically compared. According to both numerical and experimental results, the newly designed bullet exhibited greater energy transfer to the target medium and caused more extensive tissue damage, primarily due to the formation of a larger temporary cavity. These findings represent a significant advancement in understanding how bullet design and energy transfer influence wound ballistics. The newly designed bullet core produced deep and relatively flat penetration while generating a larger-diameter temporary cavity compared to the conventional 9 × 19 mm FMJ bullet, owing to its advanced nose geometry. Furthermore, since it is manufactured entirely from copper, the new bullet core is more environmentally friendly than traditional FMJ cores that typically contain lead.

The nose design is critical in achieving these effects. Radial channels incorporated into the nose geometry direct hydraulic forces inward and subsequently accelerate the fluid outward as the flow is constricted, creating high-pressure spikes that cause severe tissue damage. This rapid escalation in fluid dynamics leads to enhanced cavitation effects and extensive tissue disruption, exceeding the damage profiles observed with conventional bullets. Consequently, the newly designed bullet core achieves larger cavity formation and deep penetration without undergoing deformation or significant trajectory deviation.

Furthermore, it is important to note that the behavior of projectiles when encountering intermediate barriers remains a critical area for further research. Although this study focused on direct penetration into soft tissue simulants, ongoing experimental and numerical studies are being conducted to analyze projectile dynamics through various intermediate materials.

In conclusion, the present study developed a novel bullet core. The developed bullet core could serve as a force multiplier for law enforcement agencies by simply modifying the bullet’s internal geometry without necessitating changes to weapon calibers or incurring additional production costs.

### Key points

1. Self-defense small arms, 9×19 mm diameter weapons are most commonly used worldwide.
2. The 9×19mm Luger has not changed much since its original design.
3. A 9×19mm bullet core is redesigned to boost damage while retaining tissue penetration.
4. Tests showed the new bullet design causes more tissue damage while keeping penetration.

## Data Availability

N/A

## Code availability

N/A

## References

[1] Hogg, I. Military small arms of the 20th century. Krause Publications. 2000.

[2] Lewis Curtis, Introduction to Collecting the 9mm Parabellum (Luger) Cartridge http://cartridgecollectors.org/intro9mm/intro9mmpara.pdf (Access Date: July 22, 2021).

[3] Barnes, F. Skinner, Stan. ed. “Cartridges of the World”. 2014.

[4] Khan TH, Saha S. Numerical simulation and aerodynamic characteristic analysis of a paraboloid-tip bullet. In 4th Global Engineering, Science and Technology Conference, Bangladesh (pp. 1–8). 2013.

[5] Selimli S. Yüzey geometrisinin mermi aerodinamik davranışları üzerine etkisinin nümerik incelenmesi. Politeknik Dergisi. 2021;24(1):299–304.

[6] Widyastuti W, Wibowo AP, Pramujati B, Lesmana D, Pratama AA, Wijaya SP. Lagrangian approach embed with discrete element method for extreme deformation study in frangible bullet designs fragmentation and penetration on viscoelastic ballistic gel. Available at SSRN 2022;4269801. 10.1016/j.heliyon.2023.e14900 PMC10070894 37025784

[7] Gilson L, Rabet L, Imad A, Coghe F. Experimental and numerical assessment of non-penetrating impacts on composite protection and ballistic gelatine. Int J Impact Eng. 2020;136:103417.

[8] Riva F, Kerkhoff W, Bolck A, Mattijssen EJ. Possible influences on bullet trajectory deflection in ballistic gelatine. Forensic Sci Int. 2017;271:107–12. 28076837 10.1016/j.forsciint.2016.12.030

[9] Jin Y, Haitao L, Cheng W, Wang X, Han R, Li R, Dong D. The experimental and numerical investigation on the ballistic limit of BB—Gun pellet versus skin simulant. Forensic Sci Int. 2019;298:393–7. 30947143 10.1016/j.forsciint.2019.02.033

[10] Schyma C, Infanger C, Müller R, Bauer K, Brünig J. The deceleration of bullets in gelatine—a study based on high-speed video analysis. Forensic Sci Int. 2019;296:85–90. 30710813 10.1016/j.forsciint.2019.01.017

[11] Meng S, Taddei L, Lebaal N, Veysset D, Roth S. Modeling micro-particle impacts into ballistic gelatine using smoothed particles hydrodynamics method. Extreme Mech Lett. 2020;39:100852.

[12] Seisson G, Hebert D, Hallo L, Chevalier JM, Guillet F, Berthe L, Boustie M. Penetration and cratering experiments of graphite by 0.5-mm diameter steel spheres at various impact velocities. Int J Impact Eng. 2014;70:14–20.

[13] Ndompetelo N, Viot P, Dyckmans G, Chabotier A. Numerical and experimental study of the impact of small caliber projectiles on ballistic soap. J Phys Iv. 2006;134:385–90.

[14] Shi CC, Wang MY, Li J, Li MS. A model of depth calculation for projectile penetration into dry sand and comparison with experiments. Int J Impact Eng. 2014;73:112–22.

[15] Akers B, Belmonte A. Impact dynamics of a solid sphere falling into a viscoelastic micellar fluid. J Non-Newton Fluid. 2006;135(2–3):97–108.

[16] Frew DJ, Forrestal MJ, Cargile JD. The effect of concrete target diameter on projectile deceleration and penetration depth. Int J Impact Eng. 2006;32(10):1584–94.

[17] Sevkat E. Experimental and numerical approaches for estimating ballistic limit velocities of woven composite beams. Int J Impact Eng. 2012;45:16–27.

[18] Perdekamp MG, Pollak S, Thierauf A, Strassburger E, Hunzinger M, Vennemann B. Experimental simulation of reentry shots using a skin-gelatine composite model. Int J Legal Med. 2009;123(5):419–25. 19636582 10.1007/s00414-009-0363-6

[19] Dorogoy A, Rittel D, Brill A. Experimentation and modeling of inclined ballistic impact in thick polycarbonate plates. Int J Impact Eng. 2011;38(10):804–14.

[20] Quatrehomme G, Ǐşcan MY. Gunshot wounds to the skull: comparison of entries and exits. Forensic Sci Int. 1998;94(1–2):141–6. 9670492 10.1016/s0379-0738(98)00056-5

[21] Quatrehomme G, Işcan MY. Characteristics of gunshot wounds in the skull. J Forensic Sci. 1999;44(3):568–76. 10408112

[22] Liu GR, Quek SS. The finite element method: a practical course. Butterworth-Heinemann; 2013.

[23] Yoon GH, Mo JS, Kim KH, Yoon CH, Lim NH. Investigation of bullet penetration in ballistic gelatin via finite element simulation and experiment. J Mech Sci Technol. 2015;29:3747–59.

[24] Salisbury CP, Cronin DS. Mechanical properties of ballistic gelatin at high deformation rates. Exp Mech. 2009;49:829–40.

[25] Bui XS, Komenda J, Vítek R. Frangibility of frangible bullet upon impact on a hard target. In 2017 International Conference on Military Technologies (ICMT) (pp. 7–11). IEEE 2017.

[26] Bui XS, Vítek R, Komenda J, Skalick P. Limit impact velocity of frangible bullet. In 2019 International Conference on Military Technologies (ICMT) (pp. 1–7). IEEE 2019.

[27] Çelik E, Koç A. Analysis of free-fall bullet injury potential in the cranium via finite elements method. J Forensic Leg Med. 2023;97:102552. 37390650 10.1016/j.jflm.2023.102552

[28] Uzar Aİ, Öğünç Gİ, Özer MT. Penetran Ateşli Silah Yaralanmalarında Yara Balistiği. Güvenlik Bilimleri Dergisi, 2019;53–77.

[29] Peters CE, Sebourn CL. Wound ballistics of unstable projectiles. temporary cavity formation and tissue damage. J Trauma. 1996;40(3):16–21. 10.1097/00005373-199603001-00003 8606401

